# Heavy Chronic Ethanol Exposure From Adolescence to Adulthood Induces Cerebellar Neuronal Loss and Motor Function Damage in Female Rats

**DOI:** 10.3389/fnbeh.2018.00088

**Published:** 2018-05-15

**Authors:** Fernando B. R. da Silva, Polyane A. Cunha, Paula C. Ribera, Mayara A. Barros, Sabrina C. Cartágenes, Luanna M. P. Fernandes, Francisco B. Teixeira, Enéas A. Fontes-Júnior, Rui D. Prediger, Rafael R. Lima, Cristiane S. F. Maia

**Affiliations:** ^1^Laboratory of Pharmacology of Inflammation and Behavior (LAFICO), Institute of Health Sciences, Universidade Federal do Pará, Belém, Brazil; ^2^Laboratory of Functional and Structural Biology, Institute of Biological Sciences, Universidade Federal do Pará, Belém, Brazil; ^3^Laboratório Experimental de Doenças Neurodegenerativas (LEXDON), Department of Pharmacology, Center of Biological Sciences, Universidade Federal de Santa Catarina, Florianópolis, Brazil

**Keywords:** ethanol, adolescence, cerebellum, motor deficits, cerebellar atrophy

## Abstract

Over the last years, heavy ethanol consumption by teenagers/younger adults has increased considerably among females. However, few studies have addressed the long-term impact on brain structures’ morphology and function of chronic exposure to high ethanol doses from adolescence to adulthood in females. In line with this idea, in the current study we investigated whether heavy chronic ethanol exposure during adolescence to adulthood may induce motor impairments and morphological and cellular alterations in the cerebellum of female rats. Adolescent female Wistar rats (35 days old) were treated with distilled water or ethanol (6.5 g/kg/day, 22.5% w/v) during 55 days by gavage. At 90 days of age, motor function of animals was assessed using open field (OF), pole, beam walking and rotarod tests. Following completion of behavioral tests, morphological and immunohistochemical analyses of the cerebellum were performed. Chronic ethanol exposure impaired significantly motor performance of female rats, inducing spontaneous locomotor activity deficits, bradykinesia, incoordination and motor learning disruption. Moreover, histological analysis revealed that ethanol exposure induced atrophy and neuronal loss in the cerebellum. These findings indicate that heavy ethanol exposure during adolescence is associated with long-lasting cerebellar degeneration and motor impairments in female rats.

## Introduction

Ethanol is one of the most widely used psychoactive drugs in the world (Room et al., 2005). Some characteristics, such as the low cost, availability and easy access, contribute to high ethanol consumption among adolescents (Zarzar et al., 2012). Indeed, it has been observed a reduced sensitivity to the ethanol-induced motor impairments among adolescents, which may contribute to heavy drinking as well as the early development of alcohol dependence (Spear, 2000). Consequently, alcohol misuse during early life can result in long-lasting health problems, including central nervous system (CNS) impairments (Beenstock et al., 2011).

The cerebral cortex, hippocampus and cerebellum are brain regions vulnerable to chronic ethanol exposure, with marked reductions in their weight and volume been found among alcoholics (Harper, 2009). Moreover, it is well documented that ethanol is more deleterious to the brain during adolescence, probably because many CNS structures are under maturation, with changes ranging from molecular components to brain weight (Toga et al., 2006). In this context, previous studies have shown cerebellar atrophy and neurodegeneration following ethanol intoxication (Pierce et al., 1999; Huang et al., 2012), with the ethanol’s deleterious effects been largely influenced by the exposure period and subjects’ ages. The cerebellum, located posterior to the pons and medulla oblongata and interior to the occipital lobes of the cerebral hemisphere, is important for fine motor coordination and motor learning (Lamont and Weber, 2012). Pascual et al. (2007) showed that rats exposed to ethanol during the preadolescent and adolescent periods displayed long-term motor impairments in adulthood due to the ethanol-induced cerebellar damage.

On the other hand, epidemiological studies have shown a marked change in the ethanol consumption profile in females, with early consumption during adolescence, often in a heavy binge-drinking manner (Elliott and Bower, 2008). Brain responses after ethanol exposure appear to be distinct between females and males. There is gender-related dimorphism in brain development that may be responsible for the increased vulnerability of female brains to ethanol exposure, both in humans and laboratory animals (Bethea et al., 2002; White and Swartzwelder, 2005). However, few studies have addressed the long-lasting impact on brain structures’ morphology and function of chronic exposure to high ethanol doses in adolescent female subjects. For instance, we recently demonstrated that adult female rats administered chronically with ethanol (6.5 g/kg/day) during adolescence displayed long-term emotional and memory deficits associated with morphological and molecular alterations in the hippocampus (Oliveira et al., 2015).

In line with this idea, for the first time, in the current study we investigated whether heavy chronic ethanol exposure during late adolescence to adulthood may induce motor impairments through a battery of behavioral tasks related to cerebellar function, as well as morphological and cellular alterations in the cerebellum of female rats.

## Materials and Methods

### Animals

Twenty adolescent female Wistar rats (35 days old in the beginning of ethanol administration) were maintained in collective cages (five animals per cage) and in a climate-controlled room on a 12-h light/dark cycle (lights on at 7:00 a.m.), with food and water *ad libitum*. All procedures were approved by the Ethics Committee on Experimental Animals of the Universidade Federal do Pará (UFPA) under license number BIO-007-09 and followed the guidelines suggested by the NIH *Guide for the Care and Use of Laboratory Animals*.

### Treatments and Experimental Design

Rats (*n* = 10 per group) received orally (gavage) distilled water or ethanol (6.5 g/kg/day, 22.5% w/v) once a day, for 55 days (animals were 90 days old at the end of this treatment) always between 7 A.M. and 8 A.M. according to a procedure previously described (Teixeira et al., 2014; Oliveira et al., 2015). A daily dose of ethanol starting at 3.8–9 g/kg/day leads to a high blood alcohol level (BAL; up to 200 mg%) and blood ethanol concentration (BEC) up to 100 mg/dL in rats, upon intra-gastric administration of ethanol (Livy et al., 2003).

In order to avoid acute alcohol effects on behavior, 24 h after the last alcohol or water administration, rats were submitted to a battery of behavioral tests that were conducted between 08:00 A.M. and 14:00 P.M. (light phase) in a single day. Consisting sequentially of the open field (OF), pole, beam walking and rotarod tests, in order to assess locomotor activity, bradykinesia, balance and coordination, respectively. Then, animals were anesthetized and transcardially perfused for histopathological and immunohistochemistry evaluations. Moreover, all the behavioral, histopathological and immunohistochemistry analyses were performed by an experienced experimenter who was unaware of the experimental group of the animals tested.

### Blood Ethanol Concentration (BEC)

Blood ethanol concentration (BEC) was obtained on postnatal day 35, 60 min after a single alcohol administration in adolescent rats. Briefly, blood samples (*n* = 5 animals per group) were obtained through intracardiac pathway and stored at heparinized containers. Thereafter, 500 μL of blood sample was mixed to 500 μL of butanol (internal standard) and injected in the head space gas chromatography (GC/MS) with Flame Ionization Detector (FID) on GC-MS Varian equipment (Walnut Creek, CA, USA), model CP3800, equipped with automatic injection of samples (CombPalm Series number 1210469) for analysis. It was used a capillary column (CP WAX 52 CB, Varian), dimensions of 30 m × 0.32 mm × 0.25 μm, 100% polyethylene. Detector and injector temperatures were 250°C and 200°C, respectively.

### Behavioral Tests

Behavior was monitored through a video camera positioned above the apparatuses and the images were analyzed online, in an adjacent room, by a blinded experimenter.

#### Open Field

The OF was made of black-painted wood, with a black floor of 100 × 100 cm (divided by white lines into 25 squares of 20 × 20 cm) and 40 cm-high black walls. The experiments were conducted in a sound-attenuated room under low-intensity light (10 lx). Animals were allowed to explore the OF for 5 min. The number of squares crossed was registered as an index of general activity (Oliveira et al., 2015). A quadrant was considered crossed when the animal crossed with four paws into the adjacent square according to previous studies in our group (Teixeira et al., 2014; Oliveira et al., 2015). The apparatus was cleaned with ethanol solution (10% v/v) and dried with paper towels after each trial to avoid odor impregnation.

#### Pole Test

Bradykinesia is a characteristic signal related to cerebellar damage (Fredericks, 1996). To verify such motor profile, we employed the pole test, which consists of a sensible task designed to detect the reduced movement ability (Ogawa et al., 1985). The equipment comprises a rough vertical beam (2 × 50 cm) supported on a circular platform (1 cm height; *r* = 25 cm). Briefly, animals were placed head-upward on the top of the beam and allowed to turn upside down and move in the direction of the platform (Antzoulatos et al., 2010) in five attempts at intervals of 60 s. The escape latency (cut-off 120 s) was registered. Animals unable to conclude the task were assigned the maximum time. The three best scores were considered for each rat according to Antzoulatos et al. (2010) protocol.

#### Beam Walking

Ataxia and dystonic movements can be evaluated by beam walking test. In fact, this test has been suggested as a useful tool to motor coordination and refinement analysis (Carter et al., 1999). Motor coordination and balance in spontaneous movement were assessed by measuring the time taken to traverse a wood beam (1 m), both round (28-, 12- and 5-mm diameter) and square (28-, 17- and 11-mm diameter) shaped, elevated 50 cm of the floor (Carter et al., 1999). Initially, the rats were acclimated on the beams of higher cross-sectional area, limited to 120 s to reach the box. On the subsequent two attempts, animals were able to explore each beam (maximum of 60 s), separated by an interval of 60 s according to the modified protocol of Carter et al. (2001). Animals were allowed to reach a closed box secured (20 × 20 cm) at the end of the beams. The parameters evaluated were the time taken to complete the task and the number of slips.

#### Rotarod

The rotarod apparatus (Insight^®^, Brazil) consists of a grooved metal roller (8 cm in diameter), separated into 9 cm wide compartments elevated 16 cm. The rotarod is widely used to evaluate motor coordination of the front and hind limbs, as well as balance in rodents, which is the outcome most affected by ethanol. Ataxia resulted from cerebellar damage can be detected in the rotarod, mainly associated to beam walking test (Carter et al., 1999; Oleas et al., 2013). The rotarod test is a useful tool to evaluate gait disturbance as well as motor coordination and at higher speeds, dysdiadochokinesia (Rozas et al., 2007). The animals were evaluated for their ability to remain on the rotating rod during five successive trials of 2 min at 8 rpm with an inter-trial interval of 60 s (Teixeira et al., 2014).

### Morphometric, Histopathological and Immunohistochemistry Analyses

#### Perfusion and Tissue Procedure

Following behavioral analyses, animals in each group were deeply anesthetized with ketamine hydrochloride (90 mg/kg, i.p.) and xylazine hydrochloride (10 mg/kg, i.p.). When all corneal and paw withdrawal reflexes were abolished, rats were transcardially perfused with heparinized 0.9% saline solution followed by 4% paraformaldehyde in 0.2 M phosphate buffer. Surgical manipulation was performed only after both corneal and paw withdraw reflexes were abolished. Cerebellum was removed from the skull and post-fixed for 12 h in the same fixative. After, the cerebellum of each animal was weighed and mesoscopy measurement was made by digital pachymeter (Mitutoyo Corp., CD 56″ CT) in the antero-posterior, dorso-ventral and latero-lateral regions. Samples were subsequently cryoprotected in increasing concentrations of sucrose–glycerol solution over 7 days and then frozen in TissueTek^®^. After this 20- and 50-μm thick coronal sections of the cerebellum were cut using a cryostat (Carl Zeiss Micron, Germany) for immuhistochemistry and morphometric analyses, respectively. Sections were mounted onto gelatinized slides, air dried for 24 h and stored in a freezer at −20°C for posterior histopathological analysis.

#### Cerebellum Cellular Density Evaluation

Coronal sections of 50 μm thickness of cerebellum were stained with cresyl violet, dehydrated and coverslipped with Entellan^®^ (Merck, Germany). Illustrative images from the experimental groups were obtained with a digital camera (Moticam 2500, USA) attached to a microscope (Nikon, Eclipse 50i, USA).

#### Immunohistochemistry

Coronal sections of 20 μm thickness of cerebellum were used for immunohistochemical analysis. The immunohistochemical procedure has been described in detail in our previous studies (Lima et al., 2008; Teixeira et al., 2014; Oliveira et al., 2015). The slide-mounted sections were removed from the freezer, kept in an oven at 37°C for 30 min and rinsed in 0.1 M phosphate buffered saline (PBS) for 5 min. To improve labeling intensity, sections were treated with 0.2 M boric acid (pH 9.0) that had been previously heated to 65°C for 25 min. Sections were allowed to cool down for 20 min and incubated under constant agitation in 1% hydrogen peroxide solution in methanol for 20 min. Then, sections were rinsed three times in 0.05% PBS/Tween (Sigma Company, USA) solution for 5 min and incubated with 10% horse (NeuN) serum in PBS for 1 h.

Sections were then incubated overnight with the primary antibody in PBS, NeuN (1:500, Millipore) rinsed in PBS/Tween solution for 5 min (three times), and incubated with horse anti-mouse secondary antibody (Vector Laboratories, USA) diluted at 1:500 in PBS, for 2 h. Sections were rinsed again for 5 min (three times) and incubated in the avidin-biotin-peroxidase complex (ABC Kit, Vector Laboratories, USA) for 2 h. Sections were rinsed four times (5 min each) and developed in 3,3′-diaminobenzidine (DAB). After the DAB reaction, sections were rinsed two times (5 min each) in 0.1 M PBS, dehydrated, and coverslipped with Entellan (Merck, Germany). Illustrative images from all experimental groups were obtained with a digital camera (Moticam 2500, USA) attached to a microscope (Nikon, Eclipse 50i, USA).

#### Quantitative Analyses of Total Neu-N+ Cell Density

To evaluate mature neurons, we employed cerebellar coronal sections. Using a 1 mm^2^ graticule attached to the eyepiece (40× objective) of the microscope (Nikon, Eclipse E200), three fields were counted per section of three sections per animal, in both molecular and granular cell layers.

### Statistical Analysis

All values are expressed as mean ± SEM (*n* = 10 animals per group for behavioral tests; *n* = 5 animals per group for BEC analyses; *n* = 5 animals per group for histological evaluations). Gaussian distribution was analyzed by Kolmogorov-Smirnoff test. One-way analysis of variance (ANOVA) with repeated measures followed by *post hoc* Bonferroni’s test was applied for the rotarod data. Beam walking test was analyzed by two-way ANOVA followed by *post hoc* Tukey’s test. Statistical analysis for the rest of data was carried out using the Student’s *t*-test. Values of *P* ≤ 0.05 were considered statistically significant. GraphPad Prisma 5.0 (San Diego, CA, USA) software was used for all analyses.

## Results

### The 6.5 g/kg/Day Ethanol Paradigm Reaches High BEC Levels

Heavy ethanol administration on the 35th day of life reached a BEC of 242.6 ± 14.5 mg/dL, which reflects a high alcohol exposure in female rats.

### Effects of Chronic Ethanol Exposure During Adolescence Impair Motor Function in Female Rats

**Figure 1:**
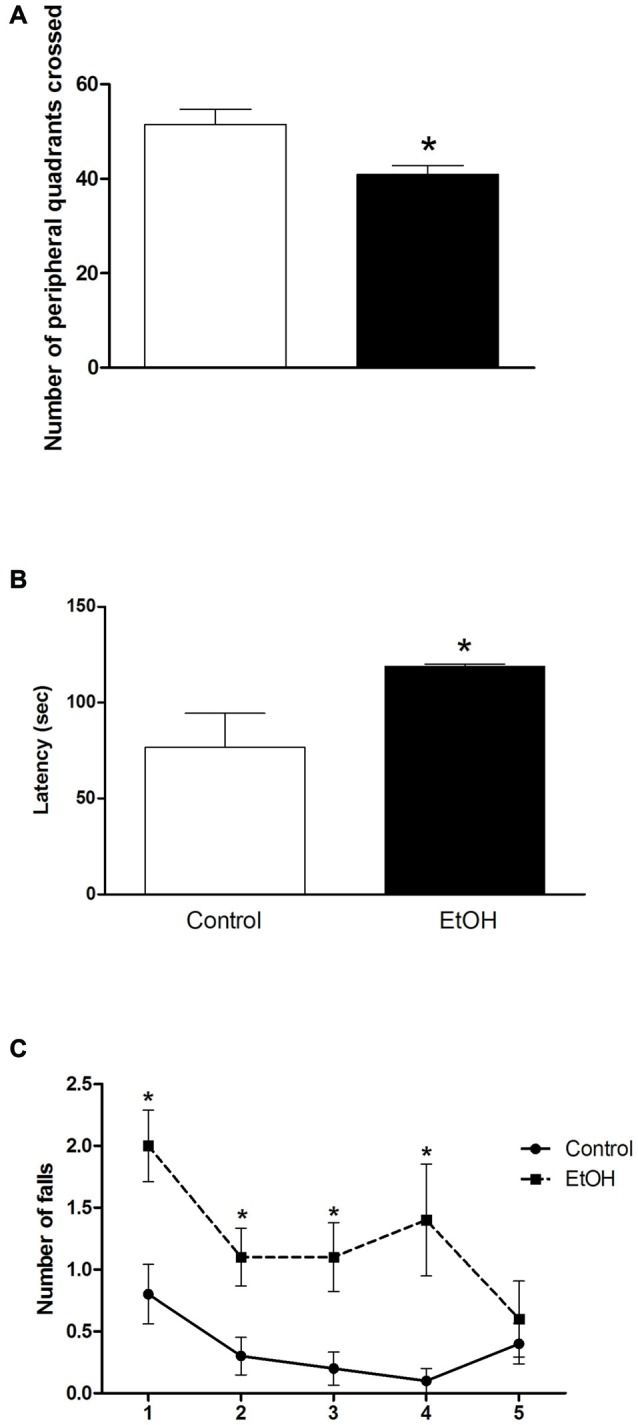
Effects of chronic ethanol (EtOH) administration (6.5 g/kg/day) during adolescence over a period of 55 days (i.e., from the 35th day until the 90th day of life) on locomotor activity of female rats in the open field (OF) test (panel **A**); on the performance in the pole test (panel **B**); and motor learning on the rotarod test (panel **C**). The results are expressed as the mean ± SEM of the number of peripheral squares crossed, latency to turn head-down and completely descend of the platform, and number of falls in five consecutive sessions (8 rpm), respectively (*n* = 10 animals per group). *p < 0.05 compared with the control group (Student’s *t*-test and one-way analysis of variance (ANOVA) with repeated measures followed by *post hoc* Bonferroni’s test for rotarod assay).

As illustrated in Figure 1A, chronic ethanol (6.5 g/kg/day) exposure over a period of 55 days induced a significant reduction on the peripheral spontaneous locomotor activity of female rats in the OF.

The results of the effects of ethanol administration during adolescence in the performance of female rats on pole test are illustrated in Figure 1B. Student’s *t*-test revealed that animals intoxicated with ethanol during adolescence displayed an increased latency to turn head-down and to descend from the platform in the pole test.

To evaluate the effect of chronic ethanol administration during adolescence on motor learning, we subjected the animals to five consecutive sessions (8 rpm) on the rotarod apparatus (Figure 1C). One-way ANOVA revealed that female rats intoxicated with ethanol increased the number of falls in the first, second, third and fourth presentation to the rotarod apparatus. The performance of the intoxicated group was like the control group only in the last session of the test (Figure 1C).

**Figure 2:**
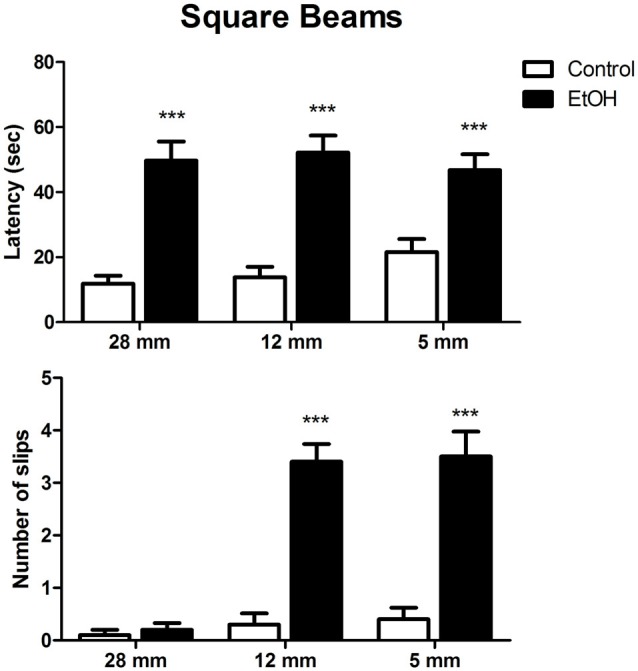
Effects of chronic EtOH administration (6.5 g/kg/day) during adolescence over a period of 55 days on motor coordination and balance of female rats addressed using square beams in the beam walking test. The results are expressed as the mean ± SEM of latency to perform the task in all diameters evaluated and the number of slips on each diameter platform (*n* = 10 animals per group). ***p ≤ 0.05 compared with the control group (Two-way ANOVA followed by *post hoc* Bonferroni’s test).

The effects of chronic ethanol exposure during adolescence in female rats on motor coordination and balance evaluated in the square beam-walking test are shown in Figure 2. Two-way ANOVA revealed that ethanol-treated rats displayed increased latency to perform the task on all beam diameters evaluated. Indeed, ethanol administration significantly increased the number of slips during the session, except for the larger beam (Figure 2).

**Figure 3:**
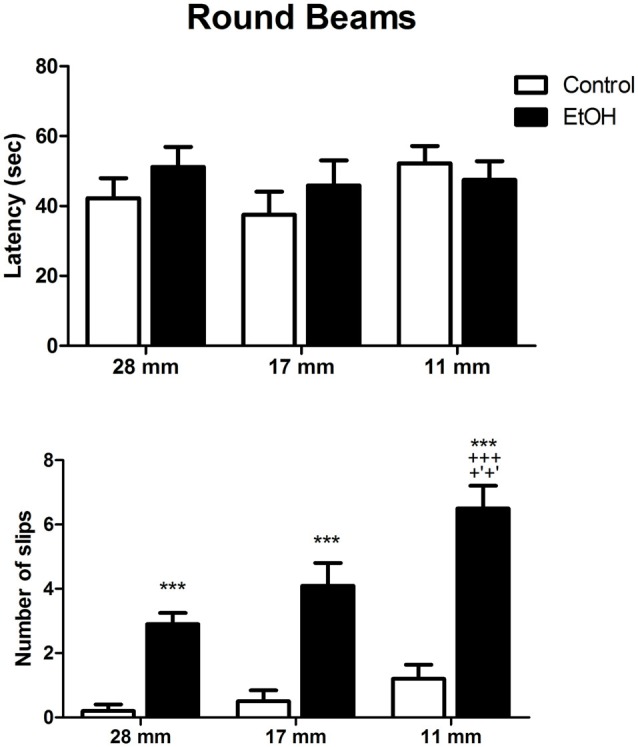
Effects of chronic EtOH administration (6.5 g/kg/day) during adolescence over a period of 55 days on motor coordination and balance of female rats addressed using the round beams in the beam walking test. The results are expressed as the mean ± SEM of latency to perform the task in all diameters evaluated and the number of slips on each diameter platform (*n* = 10 animals per group). ***p ≤ 0.05 compared to the control group on number of slips; ^+++^*p* ≤ 0.05 compared to the ethanol group in 28 mm beam; ^+′+′^*p* ≤ 0.05 compared to the ethanol group in 17 mm beam (Two-way ANOVA followed by *post hoc* Bonferroni’s test).

In order to make the task more complex, following square beam sessions, animals were tested on the round beams. Two-way ANOVA revealed that control and ethanol-treated animals spent similar times to cross the round beams. However, the number of slips per session was significantly increased by ethanol administration in all diameters evaluated (Figure 3).

### Effects of Chronic Ethanol Exposure During Adolescence on Morphological and Immunohistochemistry Parameters in the Cerebellum of Female Rats

**Figure 4:**
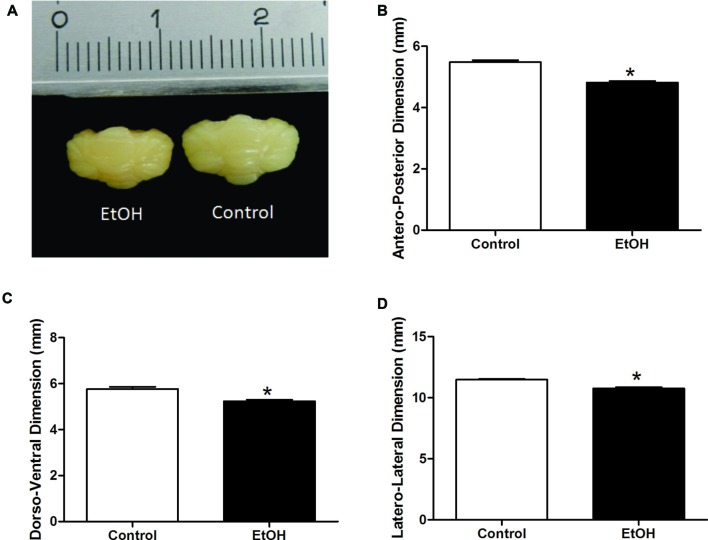
Effects of chronic EtOH administration (6.5 g/kg/day) during adolescence over a period of 55 days (i.e., from the 35th day until the 90th day of life) on cerebellar dimensions of female rats. **(A)** Represents the cerebellar macroscopic image of experimental groups. The results are expressed as the mean ± SEM of antero-posterior **(B)**, dorso-ventral **(C)** and latero-lateral **(D)** cerebellar dimensions (mm) (*n* = 5 animals per group). *p ≤ 0.05 compared with the control group (Student’s *t*-test).

Gross anatomy and histological analysis after chronic ethanol exposure in the adolescence period revealed that female rats intoxicated with ethanol displayed reduced dorso-ventral, antero-posterior and latero-lateral cerebellar dimensions, although with no changes in cerebellar mass (Figure 4).

**Figure 5:**
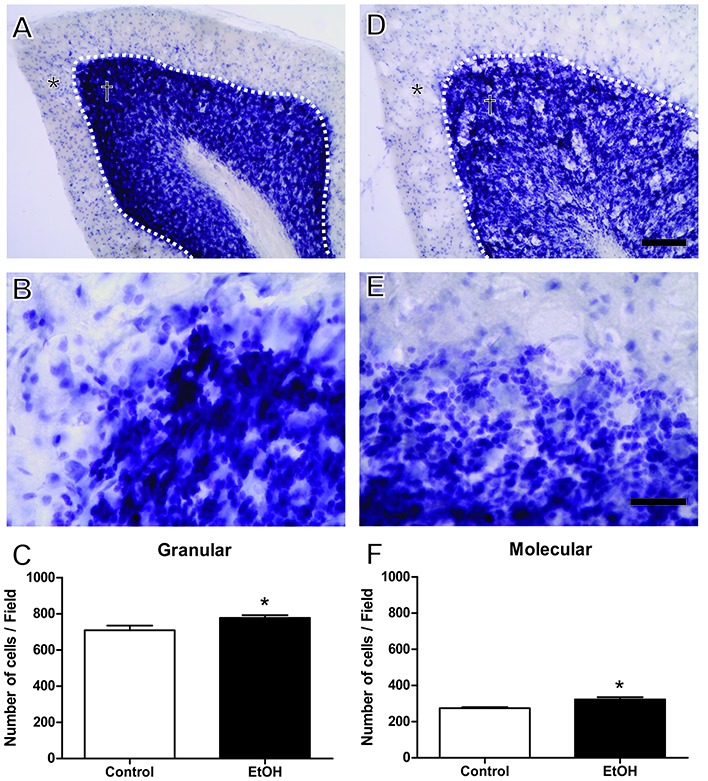
Effects of chronic EtOH administration (6.5 g/kg/day) during adolescence over a period of 55 days on number of cells on granular (†) and molecular (*) layers of the cerebellum of female rats. **(A,B)** Represent the illustrative photomicrograph of the control group. **(D,E)** Represent the illustrative photomicrograph of the EtOH group. **(C)** presents the quantitative analysis of the number of cells per field of the granular layer. **(F)** Presents the quantitative analysis of the number of cells per field of the molecular layer. The results are expressed as the mean ± SEM of the number of cells in both layers addressed with cresyl violet staining (*n* = 5 animals per group). *p < 0.05 compared with the control group (Student’s *t*-test). Scale Bar: **(A,D)** 100 mm; **(B,E)** 30 mm. Dashed line between granular and molecular layers.

Cresyl violet staining indicated a significant increase in the number of cells in the molecular layer (control 274.5 ± 5.51 vs. ethanol 322.5 ± 12.77; *p* = 0.019) and granular layer (control 709.5 ± 26.12 vs. ethanol 778.0 ± 14.72; *p* = 0.038) in the cerebellum of ethanol-treated rats (Figure 5).

**Figure 6:**
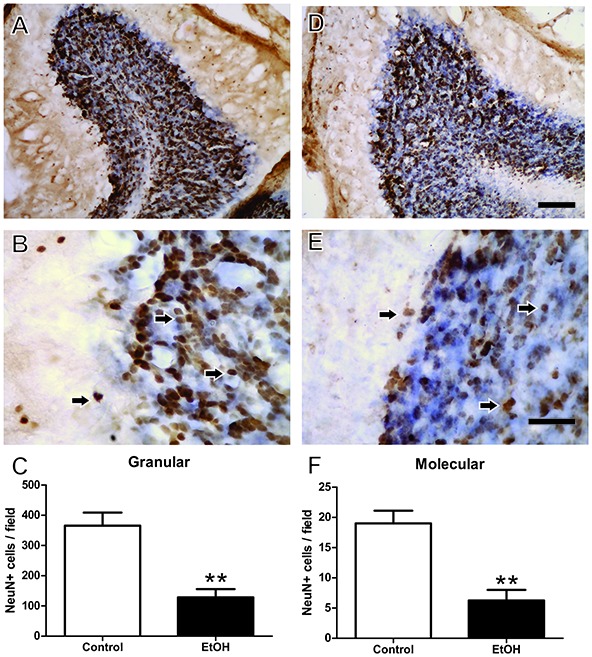
Immunohistochemistry analysis of the Effects of chronic EtOH administration (6.5 g/kg/day) during adolescence over a period of 55 days on cerebellum of female rats. **(A)** and **(B)** Represent the illustrative photomicrograph of the control group. **(D,E)** Represent the illustrative photomicrograph of the EtOH group. **(C)** Presents the quantitative analysis of the number of NeuN+ cells per field of the granular layer. **(F)** Presents the quantitative analysis of the number of NeuN+ per field of the molecular layer. The results are expressed as the mean ± SEM of the number of Neu-N+ labeled cells in the granular and molecular layers (*n* = 15 animals per group). **p < 0.01 compared with the control group (Student’s *t*-test). Scale Bar: **(A,D)** 100 mm; **(B,E)** 30 mm. Arrows indicate NeuN+ cells.

Immunohistochemistry analysis after chronic ethanol exposure during the adolescence period revealed that female rats intoxicated with ethanol presented reduced Neu-N+ labeled cells in the granular (control 365.8 ± 43.26 vs. ethanol 128.6 ± 26.99; *p* = 0.008) and molecular layers (control 19.0 ± 2.12 vs. ethanol 6.25 ± 1.75; *p* = 0.003; Figure 6).

## Discussion

The present findings demonstrate that our ethanol protocol reaches the high chronic alcohol exposure, which during adolescence in female rats induces marked long-term motor impairments evaluated through motor behavioral tasks that may reflect specific and detailed motor dysfunction, related to the direct involvement of the cerebellar modulatory function. More importantly, histological and immunohistochemistry analyses revealed that the current ethanol administration protocol induced atrophy and neuronal loss in the cerebellum of female rats.

The cerebellum is involved in the coordination, planning and fine regulation of voluntary movement as well as the control of balance, and is vulnerable to chronic alcohol exposure, of which the Purkinje cells seem to be the main target (Pierce et al., 1999). According to a comparative chronological study between human and animal models, neurochemical and neurobehavioral development in rats occurs by around the 27th to 42nd postnatal day (adolescence period; Spear, 2000, 2004). Indeed, the possibility that females mature earlier than males has been discussed (Spear, 2000; Bava et al., 2010).

Although cellular morphology and synaptic connectivity are mature by the 4th postnatal week in rodents (28 postnatal day), certain refinements of the dendritic network of Purkinje cells can happen around the end of the 3rd month (90 postnatal day; McKay and Turner, 2005). Furthermore, during adolescence, there is intense activity in the CNS for maturation to occur, making this period a window of cell vulnerability to the action of drugs such as ethanol (Spear, 2000, 2004).

In the present study, we further demonstrated that an intermittent high ethanol administration (6.5 g/kg/day, 22.5% w/v) during adolescence significantly reduced the spontaneous locomotor activity of female rats addressed in the OF. These results are in accordance with previous findings from our group showing that this same schedule of ethanol administration reduced the number of squares crossed by male rats in the OF (Teixeira et al., 2014).

Bradykinesia and decreased muscle tone can be also observed following cerebellar degeneration (Fredericks, 1996). Thus, pole test was performed to address the capacity of the animal to turn over its body axis and step down to the platform. In the present study, chronic ethanol intoxication during adolescence increased the latency of female rats to turn head-down and completely descend to the platform of the task. Purkinje cell degeneration induced by chronic ethanol exposure promotes decreased muscular tension (Peacock et al., 2014) and essential tremor (Deik et al., 2012), a cerebellar system dysfunction (Louis et al., 2011) that could justify the results observed in the pole test.

Cerebellar Purkinje cells are GABAergic neurons and consist of the single cerebellar cortex output neurons that project to the vestibular nuclei and deep cerebellar nuclei that, in turn, form the connections with the thalamus and brainstem and are involved in motor control (Kayakabe et al., 2014). Moderate doses of alcohol cause motor incoordination by potentiation of GABAergic activity at δ subunit-containing gamma-aminobutyric acid type A (GABA-A) receptors (Bowen et al., 2015). However, chronic exposure to ethanol is accompanied by changes in structure and function of GABA-A receptors, including their downregulation by increased endocytosis, and thus reducing phasic and tonic inhibition by GABAergic actions, producing motor impairments (Kayakabe et al., 2014).

In a validation study of beam walking test, the number of GABA-A receptors occupied by GABA was inversely proportional to the number of slips in the task (Stanley et al., 2005). In this sense, the beam walking test protocol becomes a sensible test for motor disorders in animals and has been broadly employed to evaluate ataxia and dystonic characteristics. Moreover, the performance of beam walking plus rotarod tests has been recommended for a better evaluation of motor coordination and refinement (Carter et al., 1999). In this sense, our results demonstrated that chronic ethanol exposure reduced motor performance in the beam walking protocol, mainly by increasing the foot slip parameter, which requires adequate motor balance, planning and coordination.

Damage to the cerebellar structures (i.e., in the Purkinje cells or in the cerebellar connections with other brain areas) promotes impairment in motor tasks, such as increases in the slip numbers and reduced latency to fall in the beam walking test and rotarod, respectively (Oleas et al., 2013). On the other hand, previous studies have demonstrated that chronic ethanol exposure produces changes in the development of climbing fibers, leading to poor innervation of Purkinje cells, resulting in significant ataxia and incoordination (Pierce et al., 2011).

The rotarod test is a useful tool to evaluate gait disturbance as well as motor coordination and at higher speeds, dysdiadochokinesia (Rozas et al., 2007). In the present study, we observed that intermittent alcohol exposure during adolescence increased the number of falls in the consecutive sessions of the rotarod, thus indicative of motor learning impairments.

We previously reported that heavy chronic ethanol exposure during adolescence until adulthood impaired motor coordination and balance, reducing latency to the first fall on the rotarod apparatus, in male rats (Teixeira et al., 2014). Moreover, Pascual et al. (2007) demonstrated that heavy drinking during adolescence/peri-adolescence increases the production of inflammatory and apoptotic mediators in the brain, which leads to the impairment of motor functions, such as poor performance in the rotarod and beam walking tests. Such mechanisms are poorly understood; however, there is a consensus that structural changes in the cerebellar circuitry occur, as well as communication between the cerebellum and other brain areas (Stanley et al., 2005; Chanraud et al., 2007; Rozas et al., 2007).

In the current study, structural modification was observed in the cerebellum, with marked cerebellar atrophy, although with no changes in cerebellar mass. In the protocol by Huang et al. (2012) in which ethanol (3.75 g/kg) was administered intraperitoneally in mice during peri-adolescence (postnatal day 25–70) or adulthood (postnatal day 180–225), the authors reported that in early adolescence, ethanol did not alter the cerebellar mass, thus corroborating our findings.

On the other hand, previous studies addressing cerebellar morphometry have revealed diminished size following chronic ethanol administration (Chanraud et al., 2007). Binge drinking during the past 3 months by 16–19-year-old subjects induced smaller bilateral cerebellar gray and white matter volumes (Lisdahl et al., 2013). Such alcohol-cerebellar effects on histological findings were attributed to the action of alcohol on GABAergic neurons and glutamatergic granule cells (Valenzuela et al., 2010), affecting Purkinje neuron plasticity (Pierce et al., 2011) and upregulating inflammatory mediators, which lead to neuronal death, atrophy and/or reduced synaptic refinement (Pascual et al., 2007).

In addition to the macroscopic effects, we investigated in the present study putative cellular alterations in the cerebellar layers. Microscopic evaluation showed that alcohol exposure during adolescence in females increased cellular density in both molecular and granule cerebellum layers. However, this alteration was accompanied by a significant reduction in Neu N^+^-labeled cells. Moreover, the present data demonstrated that early chronic ethanol exposure reduces the number of neuronal cells in the cerebellum. Similar results have already been described by our group using the same ethanol protocol in other brain areas, such as the motor cortex (Teixeira et al., 2014) and hippocampus (Oliveira et al., 2015).

Nowadays, there is a consensus that alcohol exposure alterations are dependent on dosage, frequency and period of exposure (Maier and West, 2001). The exact mechanism involved in neuronal loss is unknown; however, a study by Chuang et al. (2011) suggests that alcohol is able to interfere in the neurogenic process, with a consequent reduction of NeuN+ cells (Chuang et al., 2011). Purkinje cell death associated with chronic ethanol exposure has been related to the increased production of apoptotic factors (such as caspase-3) in the rat cerebellum (Rajgopal et al., 2003). Moreover, the proposed mechanism involves excitotoxicity via the hyperactivation of glutamate N-methyl-D-aspartate (NMDA) receptors, inducing cell granular layer degeneration (Jaatinen and Rintala, 2008), decreasing the volume of the Purkinje cell dendritic network in the molecular layer, and causing a disturbance in motor tasks (Mitra and Nagaraja, 2008).

In our results we found a decrease in neuronal density, showing that at this dose and time of exposure to ethanol in adolescence can trigger neuronal degeneration (Teixeira et al., 2014; Oliveira et al., 2015). While we observe this decrease of mature neurons in the cerebellum (NeuN+ cells), we find an increase in the total number of non-neuronal cells, which we believe to be glial cells. Glial cells, especially microglia and astrocytes, are considered the initial effector cells of the ethanol-mediated immune response, responsible for secreting inflammatory mediators and neurotoxic factors (Henriques et al., 2018). In this work, we investigated whether there was neurodegeneration in the cerebellum, but other studies may better elucidate the ethanol-mediated glial response in adolescence. All this together reflected in a poor performance on motor task.

Although it is difficult to compare the level of motor impairment displayed by ethanol consumption on the adolescence and adulthood, several studies have highlighted the called “window of vulnerability”, related to prefrontal cortex and limbic system maturation, which becomes the immature brain more susceptible to the neurotoxic effects of ethanol (Semple et al., 2013; Skala and Walter, 2013).

It is noteworthy that the acute withdrawal after a heavy chronic ethanol paradigm may contribute to the motor impairment observed in the present study, however, the cerebellum histological findings play a pivotal role and support the motor function alterations found. Our results provide new evidence that chronic exposure to high ethanol doses during adolescence promotes motor damage related to cerebellar dysfunction in female rats. The exact mechanisms involved in the observed ethanol-induced motor impairments should be investigated in future research, but the present histological and immunohistochemistry data revealed that the current ethanol administration protocol induced atrophy and neuronal loss in the cerebellum of female rats.

## Author Contributions

All authors contribute to the data analyses of the study. FBRS, PAC and CSFM: design of the work. FBRS, PAC, PCR, MAB, SCC, FBT and LMPF: acquisition of data. FBRS, PAC, RRL, EAF-J and CSFM: analysis of data. RRL, CSFM and RDP: interpretation of data. CSFM and RDP: drafting of the manuscript and critical revision of the manuscript. All authors revised and approved the final version of the manuscript.

## Conflict of Interest Statement

The authors declare that the research was conducted in the absence of any commercial or financial relationships that could be construed as a potential conflict of interest. The reviewer MCM and handling Editor declared their shared affiliation.
